# Inhaled sevoflurane use for myoclonic status secondary to bupropion intoxication

**DOI:** 10.62675/2965-2774.20250296

**Published:** 2025-06-30

**Authors:** Artur Ribeiro Canasiro, Marcelo Park, Luis Carlos Maia Cardozo, Giovanna Rego Vilar, Ludhmila Abrahão Hajjar

**Affiliations:** 1 Universidade de São Paulo Faculdade de Medicina Hospital das Clínicas São Paulo SP Brazil Intensive Care Unit, Hospital das Clínicas, Faculdade de Medicina, Universidade de São Paulo - São Paulo (SP), Brazil.

**Keywords:** Sevoflurane, Bupropion, Propofol, Midazolam, Poisoning, Myoclonus, Depression, Intensive care units

## Abstract

A 26-year-old female with a history of depression was admitted after ingesting 7.5g of bupropion. Her clinical status rapidly deteriorated into a coma and myoclonic status, which was complicated by lung aspiration. Initial treatment with high-dose midazolam and later propofol failed to control her myoclonus. Sevoflurane inhalation therapy (6.5 mg/hour) was initiated, and complete resolution of myoclonus was achieved within hours. Propofol was discontinued, and the sevoflurane dose was gradually tapered over 24 hours without myoclonus recurrence. The patient awoke agitated but neurologically intact, was extubated, and fully recovered by Day 10. This case highlights the efficacy of sevoflurane in managing refractory myoclonic status due to bupropion toxicity, especially when electroencephalogram monitoring is unavailable. Sevoflurane rapid titration and elimination allow precise sedation control and safe neurological assessment. Inhaled anesthetics may also be beneficial in other ICU scenarios, including status epilepticus, severe asthma, and hemodynamic instability. This successful outcome demonstrates the potential of sevoflurane as an alternative therapy in critical toxicological emergencies.

## INTRODUCTION

Bupropion overdose is a serious medical emergency that can lead to life-threatening neurological and cardiovascular complications, including seizures, myoclonus, and coma. Aggressive intervention is often required for managing these cases, particularly when standard therapies fail to control refractory symptoms. Here, the case of a 26-year-old female with severe bupropion intoxication who developed myoclonic status and coma is reported. Unconventional treatment with inhalational sevoflurane was required after the failure of traditional sedatives.

## CASE DESCRIPTION

**Figure 1:**
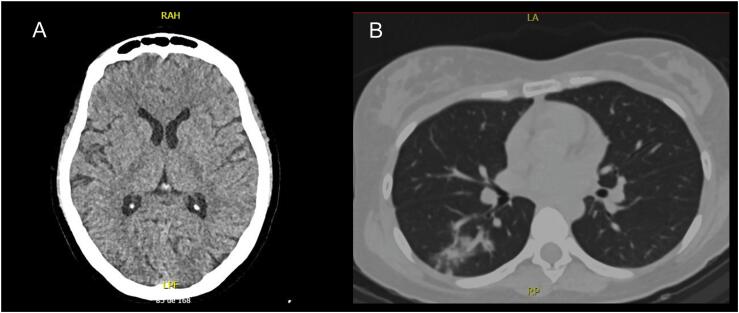
Computed tomographic images of the patient: (A) brain scan and (B) lung scan.

A 26-year-old female patient with a previous diagnosis of depression was admitted to a primary care hospital after the ingestion of 7.5g of bupropion. Her clinical status deteriorated in hours to coma and myoclonic status, complicated by lung aspiration. She was referred to our hospital. In the emergency room, she was intubated and received a continuous infusion of midazolam up to 0.7mg/kg/hour. Additional boluses of 1 - 6mg were administered, but myoclonus was not controlled. Brain and lung computed tomographic evaluation revealed consolidation in the right lung (Figure 1). No antibiotics were used. In the intensive care unit (ICU), midazolam was discontinued, and continuous infusion of propofol was initiated and titrated to 2mg/kg/hour within 12 hours. Several additional boluses of 50 - 100mg of propofol were administered but still without control of myoclonus. No additional enteral antiepileptic drugs were used. At this time, sevoflurane inhalation was initiated at 6mg/hour (via the Sedaconda ACD-S^®^ device, Sedana Medical^TM^, Sweden) and titrated to 6.5mg/hour within the first hour, achieving total control of the patient's myoclonic status. The propofol infusion was then discontinued. The laboratory results are shown in table 1.

**Table 1:** Laboratory results during the intensive care unit stay

		Admission value	Worst value*	Last measurement value
Hemoglobin (g/dL)		11.7	10.7	12.8
Leukocytes (cells/mL)		10,380	18,460	12,460
Platelets (cells/mL)		207,000	187,000	293,000
BUN (mg/dL)		7	5	8
Creatinine (mg/dL)		0.61	0.56	0.73
Sodium (mEq/L)		135	130	141
Potassium (mEq/L)		4.4	3.0	3.6
Blood gases (venous or arterial)		Venous	Venous	Arterial
	pH	7.32	7.35	7.43
	PO_2_ (mmHg)	50	44	84
	PCO_2_ (mmHg)	37	40	35
	SBE (mEq/L)	19.1	22	21
	HCO_3_ (mEq/L)	- 6.4	- 3.3	- 2.5
	SatO_2_ (%)	80.4	72	97.7
	Lactate (mMol/L)	5.3	3.3	1.7
CPK (U/L)		1,531	> 4,267	3,140
ALT (U/L)		32	38	33
AST (U/L)		38	85	75
Beta-HCG		Negative		

BUN - blood urea nitrogen; PO_2_ - partial pressure of oxygen; PCO_2_ - partial pressure of carbon dioxide; SBE - standard base excess; HCO_3_ - bicarbonate; SatO_2_ - arterial oxygen saturation; CPK - creatine phosphokinase; ALT - alanine transaminase; AST - aspartate aminotransferase; HCG - human chorionic gonadotropin.

* The worst value between admission and the last measurement.

A conventional mechanical ventilator delivered sevoflurane. When the hardware described is used, the minimum alveolar concentration of sevoflurane does not need to be monitored, nor does the end-expiratory concentration. Sevoflurane was chosen because of its cost-benefit ratio and availability at our institution.

After 24 hours, the sevoflurane dose was reduced by 1mg/hour each hour, but with an infusion of 3 mg/hour, the patient awoke agitated (Richmond Agitation-Sedation Scale 2 - 3), but without myoclonus. Sevoflurane was withdrawn without the recurrence of myoclonus; mechanical ventilation was then discontinued. Our patient progressively recovered, and at the tenth postadmission day, physical and neurological examination findings, memory, reasoning, attention, language, executive function, and social interactions were normal. She was discharged home with preplanned support.

## DISCUSSION

Bupropion is a norepinephrine-dopamine reuptake inhibitor used for major depression treatment. Bupropion overdose causes cardiovascular and central nervous system toxicity.^(1)^ Ingestion of more than 3 grams is the leading cause of induced seizures in high-income countries,^(2)^ and these seizures generally occur 30 minutes to 24 hours after oral intake, with 25% occurring beyond 8 hours after intake. More than half of patients have multiple seizures, but despite this, status epilepticus is not common.^(3)^ Interestingly, seizures can occur later and without preceding symptoms.^(4)^ Myoclonus is rare in patients with bupropion overdose^(5)^ and can be of nonepileptic origin.^(6)^ Our patient presented with very frequent myoclonic jerks. For logistical reasons, we could not obtain an electroencephalogram (EEG) in a timely manner; therefore, we cannot ascribe the coma to epileptic activity. Without an EEG, close bedside monitoring revealing improvement in awakening and myoclonus was our clinical guidance for the withdrawal of sedation. This clinical improvement was expected once the bupropion was progressively cleared. The most common methods of treating neurological manifestations of bupropion intoxication are cessation of the drug and benzodiazepine use; however, in severe cases, propofol and barbiturate use is needed.^(3)^

More severe bupropion overdoses, with doses greater than 10g are associated with severe cardiovascular manifestations such as sinus tachycardia, prolonged QTc and QRS intervals and severe cardiogenic shock,^(7)^ for which cardiovascular extracorporeal membrane oxygenation support is sometimes required.^(8)^

Inhaled anesthetics have been safe and efficaciously used in critically ill patients, with the advantages of simplicity and quick awakening.^(9)^ Furthermore, the use of inhaled anesthetics for myoclonic status control has been successfully described.^(10)^ On the basis of these described characteristics of inhaled anesthetics and the impossibility of obtaining an EEG in a timely manner, the use of sevoflurane was considered a good choice for our patient. One point to be aware of is that 5% sevoflurane is metabolized by chromosome P450 in the liver; therefore, the use of other liver-metabolized drugs must be considered with caution. Other clinical situations that could benefit from the use of inhaled anesthetics in the ICU include severe status asthmaticus, severe agitation, refractory status epilepticus, and severe hemodynamic instability.

## CONCLUSION

The quick awakening and efficient myoclonic status control of inhaled sevoflurane, delivered via a conventional mechanical ventilator, provided us with a great opportunity to safely observe and balance the control of myoclonic status and the awakening of our patient with bupropion toxicity.
